# Health Care Reform and Women’s Insurance Coverage for Breast and Cervical Cancer Screening

**DOI:** 10.5888/pcd9.120069

**Published:** 2012-10-25

**Authors:** Alice R. Levy, Brian K. Bruen, Leighton Ku

**Affiliations:** Author Affiliations: Brian K. Bruen, Leighton Ku, Department of Health Policy, George Washington University, Washington, DC.

## Abstract

**Introduction:**

The Patient Protection and Affordable Care Act of 2010 (ACA) will increase insurance coverage for US citizens and for breast and cervical cancer screening through insurance expansions and regulatory changes. The primary objective of this study was to estimate the number of low-income women who would gain health insurance after implementation of the ACA and thus be able to obtain cancer screening. A secondary objective was to estimate the size and characteristics of the uninsured low-income population and the number of women who would still need National Breast and Cervical Cancer Early Detection Program (NBCCEDP) services.

**Methods:**

We used the nationally representative 2009 American Community Survey to estimate the determinants of insurance status for women in Massachusetts, assuming full implementation of the ACA. We extrapolated findings to simulate the effects of the ACA on each state. We used individual-level predicted probabilities of being uninsured to generate estimates of the number of women who would gain health insurance after implementation of the ACA and to predict demand for NBCCEDP services.

**Results:**

Approximately 6.8 million low-income women would gain health insurance, potentially increasing the annual demand for cancer screenings initially by about 500,000 mammograms and 1.3 million Papanicolaou tests. Despite a 60% decrease in the number of low-income uninsured women, the NBCCEDP would still serve fewer than one-third of the estimated number of women eligible for services. The NBCCEDP-eligible population would comprise a larger number of women with language and literacy-related barriers to care.

**Conclusion:**

Implementation of the ACA would increase insurance coverage and access to cancer screening for millions of women, but the NBCCEDP will remain essential for the millions who will remain uninsured.

## Introduction

The percentage of American women who receive mammograms has remained steady during the past decade, whereas the percentage who receive Papanicolaou (Pap) tests has declined slightly (1). Screening and earlier treatment of breast and cervical cancer can reduce death rates (2,3), but being uninsured reduces the likelihood of screening by about half (1,4–8). A randomized experiment in Oregon demonstrated that an increase in Medicaid coverage increased the percentage of low-income women who received mammograms in the previous 12 months from 30% to 49% and increased the percentage who received Pap tests in the previous year from 41% to 58% (9,10).

In 2014, the Patient Protection and Affordable Care Act of 2010 (ACA) will expand coverage for cancer screening by reducing the number of uninsured people and by requiring private insurance and Medicare to cover breast and cervical cancer screening without cost-sharing. These insurance expansions may increase the number of women who are screened. The National Breast and Cervical Cancer Early Detection Program (NBCCEDP), which provides breast and cervical cancer screening to low-income uninsured and underinsured women, is expected to continue to be needed by millions of women who will remain uninsured. The NBCCEDP contributes to reduced breast cancer death rates (11), reduces time from cancer diagnosis to Medicaid enrollment, expands women’s treatment options (12), and changes the timing of diagnosis and treatment of cervical cancer (13,14). The primary objective of this study was to estimate the number of low-income women who would gain health insurance and thus obtain cancer screening after implementation of the ACA. A secondary objective was to estimate the size and characteristics of the population who would still need NBCCEDP services.

## Methods

### Study design

We used a simulation model based on data from Massachusetts, which enacted health reform in 2006, to estimate the effect of the ACA on women’s health insurance coverage. We used the model to estimate changes in insurance coverage among low-income uninsured women in all 50 states and the District of Columbia, assuming full implementation of the ACA.

### Health care reform nationally and in Massachusetts

The ACA is designed to increase the number of insured people by expanding Medicaid to include nonelderly adults who have an income of less than 133% of the federal poverty guidelines, by providing tax credits to use at health insurance exchanges for people who have an income of less than 400% of the federal poverty guidelines, and by requiring most people to have health insurance. These provisions are similar to those adopted in Massachusetts, which also expanded Medicaid coverage, established a state health insurance exchange, provided subsidies for people to purchase coverage in the exchange, and mandated most people to have health insurance coverage. Thus, Massachusetts provides a good case study for evaluating the national provisions (15,16). The ACA also makes regulatory changes; the following entities are required to cover preventive services recommended by the US Preventive Services Task Force, including breast and cervical cancer screening, without cost-sharing (5): qualified health insurance plans offered through health insurance exchanges, health insurance plans not designated as grandfathered, and Medicare. (Medicaid covers these services but sometimes requires copayments; Massachusetts has similar requirements.) Because of the provision of breast and cervical cancer screening without cost-sharing, virtually all women with health insurance will have affordable access to these screenings.

### Data source

The US Census Bureau’s nationally representative 2009 American Community Survey (ACS) Public Use Microdata Sample (PUMS), which includes data for 1% of each state’s population (approximately 3 million people), was our data source (17). The survey includes adults aged 18 to 64 and had a 96% response rate in 2009. From the ACS, we obtained data on health insurance status, employment status, marital and family status, age, race/ethnicity, education level, and English proficiency. The survey asked respondents to rate their ability to speak English as “only speaks English,” “very well,” “well,” “not well,” or “not at all.” We classified respondents as having limited English proficiency if they responded well, not well, or not at all.

### Statistical analysis

Because the ACA has many elements similar to those implemented in Massachusetts (15), we assumed the ACA would have an effect on individuals’ insurance outcomes comparable to what the Massachusetts reform had on outcomes for Massachusetts residents. We used a multivariate logistic regression model of the determinants of health insurance status (Appendix). The model included race/ethnicity, marital status, having at least 1 child, employment status, industry of employment, annual household income, citizenship status, disability, and education. We selected these variables on the basis of research on the determinants of health insurance coverage (18). We applied the regression coefficients of the Massachusetts equation to each individual in the ACS-PUMS sample and converted results into individual-level probabilities of being uninsured for people in all states.

Because people and institutions (eg, insurance markets) in other states may not behave like those in Massachusetts, we adjusted our estimated probabilities to reflect state characteristics. We adjusted for state policies on Medicaid eligibility for legal immigrants; Massachusetts is 1 of few states that provides Medicaid coverage (beyond emergency care) to recent legal immigrants. In addition, we used a fixed-effects model to measure the effects of other policy or market characteristics by which states differ from Massachusetts. Incorporating the state fixed effects, we calibrated our overall model to correspond to other national estimates of being uninsured developed by the Congressional Budget Office (19) and the Urban Institute (20). Finally, we created adjusted 2014 weights to reflect expected changes in the size and age distribution of the population from 2009 to 2014 on the basis of Census Bureau population projections.

By using our adjusted predicted probabilities of being uninsured and adjusted population weights, we derived state-level predicted probabilities and population sizes. We generated estimates for low-income women aged 40 to 64 (the target range for NBCCEDP breast cancer screening) and 18 to 64 (the target range for NBCCEDP cervical cancer screening). The US Preventive Services Task Force recently changed its guidelines to recommend cervical cancer screening beginning at age 21 (21), so NBCCEDP target ranges may change as well. We defined “low-income” according to each state’s income eligibility limits, which ranged in 2011 from 185% to 250% of the federal poverty guidelines: 185% in Oklahoma; 200% in Alabama, Arkansas, California, Connecticut, Florida, Georgia, Idaho, Indiana, Missouri, Montana, North Dakota, Ohio, South Carolina, South Dakota, Texas, Virginia, West Virginia; 225% in Kansas and Nebraska; and 250% in all other states. We computed changes in insurance coverage by comparing 2009 data with estimates for 2014. To derive national estimates, we summed state estimates. We grouped states into quartiles according to the uninsured rate projected for 2014.

We used results from the recent Oregon Medicaid randomized experiment (9,10) to estimate the effect of increased insurance coverage on cancer screening. In the Oregon study, the number of women among those who gained insurance and who had a mammogram within a year was 18.7% higher than the number in the uninsured reference group. If this increase is sustained, 39% more women in the insured group than the uninsured group would have a mammogram within the recommended 2-year period. Also in the Oregon study, the number of women who had a Pap test in the past year was 18.3% higher among those who gained insurance than among women who did not. About 55% more women would be tested within the recommended 3 years if the increase was sustained. The Oregon model assumed that the increased demand for screening caused by insurance expansions could be met by the existing capacity (eg, clinicians, facilities) to provide services. We made the same assumptions. We assessed the number of women served by NBCCEDP from 2007 through 2009. Because the recommended frequency of mammograms is every 2 years for asymptomatic women and the recommended frequency of Pap tests is every 3 years (2,3), we assessed the number of women served by NBCCEDP in 2-year (2008 and 2009) and 3-year (2007 through 2009) intervals. We analyzed all data using Stata version 11 (StataCorp LP, College Station, Texas).

## Results

Nationally, approximately 6.8 million low-income women aged 18 to 64 will gain health insurance coverage as the result of the ACA. The number of uninsured low-income women aged 18 to 64 will decline by 60% — from 11.3 million to 4.5 million; the uninsured rate among these women will decline from 33.6% in 2009 to 12.9% in 2014 (Table 1). Approximately 2.8 million low-income women aged 40 to 64 will gain health insurance as the result of the ACA. The number of uninsured low-income women aged 40 to 64 will decline by 62% — from 4.5 million to 1.7 million; the uninsured rate among these women will decline from 31.1% in 2009 to 11.2% in 2014.

**Table 1:** Estimated ACA-Related Changes in Insurance Coverage and Cancer Screenings Among Low-Income Women Aged 18 to 64, 2009–2014

Variable	Women Aged 40–64^a^	Women Aged 18–64^b^
Uninsured women in 2009, actual, in thousands, n (%)	4,514 (31.1)	11,266 (33.6)
Uninsured women in 2014, projected, in thousands, n (%)	1,705 (11.2)	4,470 (12.9)
Increase in insured women from 2009 to 2014, projected, in thousands, n	2,809	6,796
Women screened by NBCCEDP, 2007–2009^c^, actual, in thousands, n	518	783
Projected annual increase in cancer screenings due to increased insurance coverage in 2014, in thousands, n	500	1,300
NBCCEDP-eligible women projected to be screened in 2014, %	30.3	17.5

Abbreviations: ACA, Patient Protection and Affordable Care Act of 2010; NBCCEDP, National Breast and Cervical Cancer Early Detection Program.

^a^ Age group recommended for breast cancer screenings.

^b^ Age group recommended for cervical cancer screenings.

^c^ Includes women screened for breast cancer in 2008 and 2009 and women screened for cervical cancer from 2007 through 2009. These values reflect the US Preventive Services Task Force recommendations of screening for breast cancer every 2 years and cervical cancer every 3 years.

During 2008 and 2009, approximately 518,000 women obtained breast cancer screening through the NBCCEDP, which is 30.3% of the 1.7 million women projected to be uninsured and eligible for NBCCEDP services in 2014. From 2007 through 2009, approximately 783,000 women obtained cervical cancer screening through the NBCCEDP, which is 17.5% of the 4.5 million women projected to be uninsured and eligible for NBCCEDP services in 2014. Despite significant increases in insurance coverage, the number of uninsured low-income women after enactment of ACA will be 3 to 5 times higher than the number now being served by the NBCCEDP. We estimated that the increase in demand for breast cancer screening will increase by an additional 500,000 women in the first year of ACA implementation and by as many as 1 million more over 2 years. Similarly, we estimate that an additional 1.3 million women will obtain a Pap test in the first year, and as many as 3.8 million more will be tested over 3 years.

The percentage of low-income women aged 18 to 64 projected to be uninsured in 2014 varied by state, from 7.8% in Maine to 19.3% in Nevada (Table 2). In California, 15.0% of these women were projected to be uninsured; in Florida, 17.3%; and Texas, 19.0%. Six of the 13 states with uninsured rates higher than 12.5% were in the Southwest (Figure).

**Table 2:** Estimated ACA-Related Changes in Uninsured Rates and NBCCEDP Cervical Cancer Screenings Among Low-Income Women Aged 18 to 64, by State, Assuming Full Implementation, 2009–2014

State	Uninsured Rate in 2009, %	Uninsured Rate in 2014, %	Uninsured in 2009, n, in Thousands	Uninsured in 2014, n, in Thousands	Increase in Insured from 2009 to 2014, n, in Thousands	Women Screened by NBCCEDP 2007–2009, n, in Thousands	Ratio of Women Screened by NBCCEDP 2007–2009 to Eligible Women in 2014, %
United States	33.6	12.9	11,266,275	4,469,633	6,796,642	782,946	17.5
Alabama	34.6	10.1	196,191	59,129	137,062	11,250	19.0
Alaska	39.4	12.4	23,175	7,489	15,686	34,134	455.8
Arizona	33.0	16.6	279,049	144,546	134,503	11,419	7.9
Arkansas	40.8	10.7	142,700	38,451	104,249	6,329	16.5
California	38.5	15.0	1,394,554	557,784	836,770	31,672	5.7
Colorado	35.7	15.6	191,072	85,981	105,091	21,396	24.9
Connecticut	21.9	10.7	50,135	25,127	25,008	7,164	28.5
Delaware	20.3	9.8	20,067	10,047	10,020	6,130	61.0
District of Columbia	10.4	8.6	8,163	7,001	1,162	453	6.5
Florida	44.0	17.3	851,097	344,971	506,126	17,494	5.1
Georgia	42.3	13.6	455,358	150,880	304,478	14,734	9.8
Hawaii	13.7	9.7	16,962	12,340	4,622	1,908	15.5
Idaho	39.3	12.1	65,399	20,684	44,715	5,580	27.0
Illinois	31.4	14.2	458,639	214,359	244,280	35,955	16.8
Indiana	33.7	11.4	214,202	74,536	139,666	12,458	16.7
Iowa	22.0	9.6	71,097	31,786	39,311	9,696	30.5
Kansas	33.1	12.5	97,680	37,980	59,700	8,557	22.5
Kentucky	34.1	9.4	214,871	60,944	153,927	25,066	41.1
Louisiana	38.3	10.4	253,201	70,949	182,252	8,638	12.2
Maine	18.2	7.8	30,358	13,430	16,928	5,654	42.1
Maryland	28.1	13.3	140,316	68,363	71,953	14,006	20.5
Massachusetts	8.5	8.9	49,359	53,330	−3,971	9,745	18.3
Michigan	26.4	10.0	347,353	136,063	211,290	37,226	27.4
Minnesota	20.1	9.3	103,185	49,053	54,132	20,396	41.6
Mississippi	32.5	8.9	152,870	43,150	109,720	9,223	21.4
Missouri	34.2	9.9	209,091	62,365	146,726	14,587	23.4
Montana	38.5	11.3	40,430	12,187	28,243	7,148	58.7
Nebraska	32.3	10.3	58,705	19,311	39,394	15,094	78.2
Nevada	44.1	19.3	140,938	63,687	77,251	12,746	20.0
New Hampshire	27.2	10.4	32,523	12,859	19,664	6,459	50.2
New Jersey	32.2	16.4	233,594	122,961	110,633	24,156	19.6
New Mexico	39.8	14.7	114,801	43,791	71,010	20,612	47.1
New York	22.1	11.1	493,258	256,070	237,188	77,612	30.3
North Carolina	33.6	12.1	434,116	160,927	273,189	19,915	12.4
North Dakota	28.5	10.4	14,698	5,486	9,212	4,092	74.6
Ohio	29.1	10.3	333,778	121,385	212,393	19,324	15.9
Oklahoma	42.5	12.3	161,259	48,162	113,097	11,494	23.9
Oregon	37.8	14.0	189,810	72,516	117,294	13,125	18.1
Pennsylvania	21.0	9.3	292,764	134,435	158,329	9,266	6.9
Rhode Island	23.5	13.0	25,538	14,552	10,986	7,220	49.6
South Carolina	37.7	11.6	202,633	64,599	138,034	18,850	29.2
South Dakota	34.8	10.3	27,513	8,390	19,123	7,470	89.0
Tennessee	29.9	10.3	271,395	96,590	174,805	13,233	13.7
Texas	52.2	19.0	1,416,540	531,716	884,824	33,385	6.3
Utah	29.4	11.9	96,233	39,998	56,235	4,778	11.9
Vermont	13.2	8.5	8,743	5,762	2,981	2,317	40.2
Virginia	32.4	12.3	196,844	77,239	119,605	7,345	9.5
Washington	30.6	10.9	227,443	83,861	143,582	28,911	34.5
West Virginia	35.5	10.0	76,359	22,123	54,236	23,420	105.9
Wisconsin	18.8	9.9	118,154	63,942	54,212	12,785	20.0
Wyoming	39.2	10.9	22,062	6,345	15,717	1,319	20.8

Abbreviations: ACA, Patient Protection and Affordable Care Act of 2010; NBCCEDP, National Breast and Cervical Cancer Early Detection Program.

**Figure:**
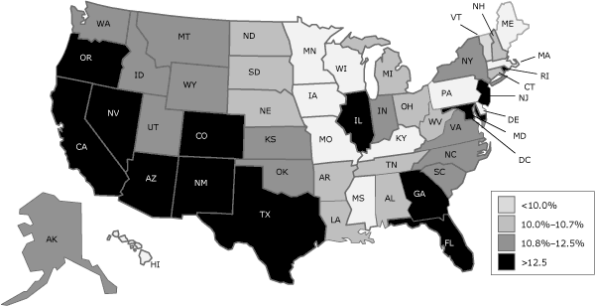
Grouping of 50 states and the District of Columbia by quartile according to percentage of low-income women aged 18 to 64 projected to be uninsured in 2014 after full implementation of the Patient Protection and Affordable Care Act of 2010. **Table:** 

<10.0%	10.0%–10.7%	10.8%–12.5%	>12.5%
Delaware, District of Columbia, Hawaii, Kentucky, Iowa, Maine, Massachusetts, Minnesota, Mississippi, Missouri, Pennsylvania, Vermont, Wisconsin	Alabama, Arkansas, Connecticut, Louisiana, Michigan, Nebraska, New Hampshire, North Dakota, Ohio, South Dakota, Tennessee, West Virginia	Alaska, Idaho, Indiana, Kansas, Montana, New York, North Carolina, Oklahoma, South Carolina, Utah, Virginia, Washington, Wyoming	Arizona, California, Colorado, Florida, Georgia, Illinois, Maryland, Nevada, New Jersey, New Mexico, Oregon, Rhode Island, Texas

The demographic characteristics of low-income women who will remain uninsured were projected to change in 2014 (Table 3). They were projected to consist of a larger percentage of Asians and Hispanics and a smaller percentage of non-Hispanic whites and blacks. A larger percentage of uninsured women in 2014 may have literacy-related barriers and limited English proficiency or lack a high school degree.

**Table 3:** Characteristics of Uninsured Women Aged 18 to 64, 2009 and 2014^a^

Characteristic	Uninsured Women in 2009, %	Uninsured Women in 2014, %
Hispanic	32.8	39.2
Black	16.3	12.8
Non-Hispanic white	44.0	39.4
American Indian	1.3	0.9
Asian	4.1	6.1
Limited English proficiency^b^	25.3	33.2
No high school degree or GED	28.5	32.6
College graduate	8.1	8.6
Child aged 1–6 at home	20.3	14.8
Child aged 7–17 at home	29.3	23.4
Receive public income	5.0	3.2
Disabled	9.8	7.7
Employed	49.8	49.1

Abbreviation: GED, general equivalency degree.

^a^ Based on data collected through the 2009 American Community Survey Public Use Microdata Sample (17).

^b^ Survey asked respondents to rate their ability to speak English as “only speaks English,” “very well,” “well,” “not well,” or “not at all.” We classified respondents as having limited English proficiency if they responded well, not well, or not at all.

## Discussion

Our model indicates that millions more low-income women will gain health insurance coverage after implementation of the ACA, which should lead to increases in levels of cancer screening among this population. The increase in screening among newly insured women should help to improve the national screening rate, which has remained steady (mammograms) or declined slightly (Pap tests) for a decade (1). Although the ACA will reduce the number of uninsured women, the NBCCEDP will still be needed to support access for millions of women who will remain uninsured. If future numbers of women served by NBCCEDP are comparable to recent numbers, the program will still only be able to meet the needs of one-fifth to one-third of those eligible. Like many public health programs, NBCCEDP is a grant program, and its funding is limited by federal and state appropriations; the program has never had sufficient funds to serve all eligible women.

This study has several limitations. While forecasts are useful, they are necessarily based on assumptions. One such assumption is that past trends can predict future trends. We assumed that the ACA will be implemented in 2014 and that its national effect will be similar to the effect of the Massachusetts reform. Even if the ACA is fully implemented as passed, complete implementation of the insurance expansions may take more time. Our model assumed that economic and social circumstances (eg, employment, income) in 2014 will be similar to those in 2009. Our projections also relied on survey data, which introduces the potential for measurement and respondent recall errors.

Our model differed from the insurance simulation models developed by the Congressional Budget Office (22) and the Urban Institute (23). These complex models are designed to compare national budgetary effects of alternative policies and are based on an amalgam of sources, particularly the Current Population Survey and the Medical Expenditure Panel Survey. These models require many submodels and assumptions about behavioral responses to various policies; models that have only slightly different assumptions may yield different results (24,25). Our model used a larger sample (the ACS) and a more transparent set of assumptions. However, our results still depended heavily on our assumptions.

We focused on uninsured women because we anticipate that almost no insured women will be underinsured for breast or cervical cancer screening after implementation of the ACA, which requires screening coverage without cost-sharing. However, some insured women may be eligible for NBCCEDP services for diagnostic tests, such as biopsies or other imaging, which may be subject to deductibles or copayments even after implementation of the ACA. Diagnostic tests are used for further assessment of abnormal screening results and for women who have a prior history of cancer. Thus, while almost no insured women should be underinsured for screening after implementation of the ACA, some may be underinsured for diagnostic testing purposes, and these women could receive free care through the NBCCEDP.

Our analyses indicate 3 shifts in the population of women who will remain eligible for NBCCEDP services. First, the geographic distribution of low-income uninsured women eligible for services will change, possibly prompting changes in the allocation of funds among states or the location of services within states. This shift will occur because the number of women projected to gain insurance varies by state. Generally speaking, the largest gains in insurance coverage were projected in states with lower per capita incomes and lower 2009 Medicaid eligibility standards for nonelderly adults. Conversely, states with more generous current Medicaid eligibility standards for adults and higher per capita incomes were projected to have smaller gains in insurance coverage. Thus, in addition to providing health insurance for millions of low-income women, health reform will change the distribution of the remaining uninsured population.

A second major shift will occur because the remaining population of eligible women will include a higher percentage of women who have a limited education and limited English proficiency. Local programs may need to adapt their educational and outreach approaches to meet the needs of these women. NBCCEDP providers should be able to provide language assistance to women who have limited English proficiency; such assistance is required under federal policy (26). Many women who have limited English proficiency are immigrants, who are an important population for public health screening because they are less likely to obtain cancer screening than other women (27,28). The third major shift is that the remaining population of NBCCEDP-eligible women will have a greater percentage of Hispanic and Asian women, the very women least likely to obtain regular breast and cervical cancer screening (1). In addition, the women who will remain uninsured despite large increases in coverage may be harder to reach with health-related messages. Lack of awareness (health literacy) (29) and transportation/geographic access (30) are barriers to cancer screening. These barriers are likely to be relevant for a larger share of the women who will remain uninsured women after implementation of the ACA.

One option to consider is to increase the percentage of NBCCEDP funds that can be spent on cancer outreach and patient navigation services, which is now capped at 40%. Currently, 60% of program funds must be spent on screening and diagnostic services and on referrals for treatment. Many more low-income women in 2014 will have access to screening through insurance coverage, so it may be appropriate to dedicate a larger share of program funds to outreach and navigation so that women, whether insured or uninsured, receive encouragement and assistance to be screened. Given that the NBCCEDP-eligible population is expected to be harder to reach with health care messages, such efforts may be critical to ensuring the program reaches its target population. This option would also help insured women who may be unaware of the need for cancer screening or who may face other barriers. Because 80% of unscreened women who have access to health care report not receiving a recommendation for a mammogram (31), there is clearly a need for additional education efforts.

Another option is to expand eligibility guidelines to include higher income levels. In addition to promoting cancer screening among moderate-income uninsured women, this policy option would help women who need diagnostic tests after screening indicates an abnormality. The NBCCEDP provides diagnostic tests without cost-sharing (whereas the ACA eliminates cost-sharing only for cancer screening services). If low- and moderate-income women are unable to afford cost-sharing for diagnostic services after receiving an abnormal screening result, they may not receive early treatment.

Millions of American women do not get screened for breast or cervical cancer. The ACA offers an opportunity to increase screening, early detection, and treatment of these cancers. Our analyses indicate insurance coverage will increase for low-income women. The Oregon trial (9,10) showed that simply increasing insurance coverage can boost screening rates for breast and cervical cancer. Despite this encouraging news, millions of low-income women will remain uninsured after implementation of the ACA. The NBCCEDP will continue to play a role in helping to ensure that low-income uninsured women have access to cancer screening services, but it may need to adapt its policies to meet new programmatic needs.

This analysis was conducted and written before the Supreme Court’s June 2012 decision to give states the option to not expand Medicaid. Several governors have since announced they will not expand Medicaid, but how many states will not implement the expansion remains unclear. For states that expand Medicaid, our state-specific estimates should be reasonable approximations of uninsured rates, but in states that do not, future uninsured rates will likely be between our estimates and their 2009 baselines. Other elements of the ACA, such as health insurance exchanges, tax credits, and the requirement to purchase insurance or pay a tax penalty, will still lead to gains in women’s insurance coverage, although the poorer women could still be denied Medicaid. If some states are also able to block the health insurance exchanges, their insurance rates should remain closer to their 2009 baselines. Because some of the states most likely to resist Medicaid expansion have higher uninsured levels, the optional nature of the Medicaid expansion means that national gains could be much lower than anticipated, as recent estimates of the Congressional Budget Office indicate (32). The number of future uninsured low-income women will depend on state policy choices and will remain high in states that fail to implement Medicaid expansions. In those states, the demand for screening services under NBCCEDP will increase.

## Appendix. Methodology for Estimating the Effect of Health Reform on Women’s Insurance Coverage and Breast and Cervical Cancer Screening

This appendix is available for download as a Microsoft Word file.
