# Erratum, Vol. 9, October 25 Release

**DOI:** 10.5888/pcd9.120069e

**Published:** 2012-11-21

Because of editing errors, we incorrectly characterized the effect of health care reform on cancer screenings under the National Breast and Cervical Cancer Early Detection Program (NBCCEDP) in the following article: “Health Care Reform and Women’s Insurance Coverage for Breast and Cervical Cancer Screening.” The first sentence of the Results section now reads “Approximately 6.8 million low-income women would gain health insurance, potentially increasing the annual demand for cancer screenings initially by about 500,000 mammograms and 1.3 million Papanicolaou tests.” In the Results section, 2 sentences were corrected: “We estimated that the increase in demand for breast cancer screening will increase by an additional 500,000 women in the first year of ACA implementation and by as many as 1 million more over 2 years. Similarly, we estimate that an additional 1.3 million women will obtain a Pap test in the first year, and as many as 3.8 million more will be tested over 3 years.” The title for Table 1 now reads “Estimated ACA-Related Changes in Insurance Coverage and Cancer Screenings Among Low-Income Women Aged 18 to 64, 2009–2014.” A row heading in Table 1 was corrected to “Projected annual increase in cancer screenings due to increased insurance coverage in 2014, in thousands, n.” The title for Table 2 now reads “Estimated ACA-Related Changes in Uninsured Rates and NBCCEDP Cervical Cancer Screenings Among Low-Income Women Aged 18 to 64, by State, Assuming Full Implementation, 2009–2014.”

The corrections were made to the article on November 2, 2012, and appear online at http://www.cdc.gov/pcd/issues/2012/12_0069.htm. We regret any confusion or inconvenience these errors may have caused.

