# Supplementary material for: Health Care Reform and Women’s Insurance Coverage for Breast and Cervical Cancer Screening
Source: Prev Chronic Dis. 2012 Oct 25;9:E159. doi: 10.5888/pcd9.120069 (PMC3499983; doi:10.5888/pcd9.120069)
Supplement: Supplementary file 1 [file 12_0069_01.doc]

**Appendix. Methodology for Estimating the Effect of Health Reform on Women’s Insurance Coverage and Breast and Cervical Cancer Screening**

This technical appendix provides additional details about our methodology for estimating the number of people who are uninsured in 2014 after the implementation of health insurance expansions under the Patient Protection and Affordable Care Act of 2010 (ACA) and for estimating 2014 eligibility for the Centers for Disease Control and Prevention’s National Breast and Cervical Cancer Early Detection Program (NBCCEDP). We focused on low-income women aged 18 to 64 years (the eligible age range for cervical cancer screening) or 40 to 64 (the eligible ages for breast cancer screening).

**Data**

The data used in the analysis come from the 2009 American Community Survey (ACS) Public Use Microdata Sample (PUMS) which includes observations on 2,979,656 people living in households in all counties and county equivalents in the 50 US states and the District of Columbia. (The PUMS sample also includes 83,238 people living in group quarters, but we dropped them from our analysis because family income is not available for this population.) These data represent a subset of all responses to the 2009 ACS: the survey as a whole aims to capture 2.5% of each state’s population, but the PUMS includes only 1% of each state’s population. Even so, the use of ACS data represents a major increase in the sample size and expected precision of state estimates over prior survey data such as the Current Population Survey.

Most existing research on health insurance status draws from other surveys — particularly the Annual Social and Economic Supplement (ASEC) to the Current Population Survey (CPS). The ACS only began including a question about health insurance status in 2008. Despite its limited prior use as a data set for modeling the determinants of health insurance status, the ACS has several advantages over the ASEC-CPS for our purposes. Most importantly, with a sample size roughly 30 times that of the ASEC-CPS and a response rate of 96% (compared to 84% for the CPS), the ACS PUMS is a much larger data set than the ASEC-CPS. For this reason, the Census Bureau recommends using the ACS for studies examining state-level outcomes (1).

In addition to its larger sample size, a major advantage of the ACS is that its health insurance status question is relatively easy to interpret. For each member of the household, the question simply asks if the individual currently has insurance through a) a current or former employer or union, b) direct purchase, c) Medicare, d) Medicaid or other government assistance, e) TRICARE or other military, f) the Veterans Administration (VA), or g) Indian Health Service. For our purposes of evaluating insurance status, we treated TRICARE or other military and VA coverage as private insurance and Indian Health Services as uninsured (if the only coverage a person had). This method of asking about health insurance results in transparent data that reflect current insurance status and facilitates the identification of insurance status for respondents’ household members. A study for the Census Bureau evaluated the health insurance data from the ACS in comparison to data from the ASEC-CPS and the National Health Interview Survey (NHIS) and found a high level of consistency for the ACS estimates (2).

We downloaded the ACS PUMS data from the Census Bureau website and read them into SAS. We transferred files to Stata using StatTransfer and performed all data recoding and calculations in Stata. Because the PUMS data is designed for public use, the Census bureau completes a significant amount of data cleaning and imputation while preparing the data. The Census Bureau edits survey responses using hot-deck and cold-deck allocation to correct for internal inconsistencies and/or item non-response prior to PUMS release.

**Research Design and Methods**

Our underlying approach was to first estimate determinants of insurance status in Massachusetts in 2009, based on the ACS data. Massachusetts implemented a state-level health insurance reform in 2006 and many of its features were used in developing the national reform, so it is reasonable to use Massachusetts results to understand effects at the national level. We developed a Massachusetts health insurance model to simulate health reform at the national level, with some adjustments. This approach assumes that individual-level determinants of health insurance for Massachusetts residents in 2009 will be similar to those for all US residents after ACA expansions are implemented in 2014. Rather than run separate models predicting Medicaid and private health insurance coverage, we parsimoniously estimated a model on being insured or uninsured, regardless of source of coverage. It is known that Medicaid coverage influences private health insurance coverage and vice versa. The availability of coverage for breast and cervical cancer screening coverage will be the same whether a person is covered by private insurance or Medicaid under the ACA.

We estimated a multivariate logit model of the determinants of health insurance status for adults aged 18 to 64 in Massachusetts in 2009, using the ACS sample weights. (A similar model was developed for men, some of whom are eligible for CDC’s colorectal cancer screening program, but those data are not reported in this article. The results of that model and estimates of male uninsured rates are available from the authors.) Our model includes race/ethnicity, marital status, having a child, employment status, industry, income, citizenship status, disability and education. We selected variables based on prior research about the determinants of health insurance coverage (3).

To capture information about the type of industry in which people are employed, a known determinant of health insurance status, we created high, average, and low health insurance industry categories. High insurance industries include utilities, finance and insurance, management of companies and enterprises, educational services, and public administration; all have uninsurance rates below 10%. Low insurance industries include agriculture, forestry, fishing, and hunting, construction, administrative support and waste management and remediation, accommodation and food services, and other services (except public administration); each of these has uninsurance rates greater than 25%. While the ACS data do not indicate which individuals were offered employer-sponsored insurance or the level of premiums charged, our industry groupings provide some control for the likelihood of health insurance offers and premium levels, on the basis of the industry in which each individual is employed (if any). Variable selection was based on theoretical reasons and predictive capabilities rather than parsimony; hence, some insignificant variables remain in the model.

The results of the multivariate logit model for predicting whether nonelderly women in Massachusetts are uninsured are shown in Table A1. We express the results as odds ratios and 95% confidence intervals, although the results were operationalized as coefficients and predicted probabilities. The following factors are associated with a significantly lower probability of being uninsured: being African American, being below poverty, higher income, working full-time full-year, being married, having a child, being disabled, receiving public income (eg, welfare), being a college graduate, being a student, and working in a high-insurance industry. Factors associated with increased uninsurance include being a noncitizen immigrant, working in a low-insurance industry, being over 39 and Hispanic (interaction) and being black and poor (interaction). Table A2 illustrates the performance of the logit model by comparing predicted uninsured rates for subgroups of Massachusetts women to actual uninsured rates for these same groups.

The regression coefficients of the Massachusetts equation were applied to each individual in the ACS-PUMS sample and converted into individual-level probabilities of being uninsured for each person in every US state. We recognized that adjustments are needed however, because 1) people in other states might not behave like those in Massachusetts, even after controlling for individual characteristics, 2) institutions and markets (eg, Medicaid agencies, insurance markets) in other states may behave differently, and 3) the Massachusetts reforms are very similar to, but not identical, to those of the ACA (although the final forms of ACA implementation are yet to be determined).

Our first adjustment concerned citizenship status, a major determinants of insurance coverage (4). Massachusetts is one of a minority of states providing state-funded Medicaid coverage to recent legal immigrant adults, so noncitizen immigrants have a higher probability of being insured than in states without such coverage. To account for the lower likelihood of insurance coverage among immigrants in less generous states, we doubled the coefficient for noncitizen status in those states not offering Medicaid to recent immigrants. These states were all except California, Connecticut, Delaware, the District of Columbia, Hawaii, Maine, Massachusetts, Minnesota, Nebraska, New York, and Washington. Data on Medicaid policy on immigrants were obtained from the National Immigration Law Center (5).

To adjust state estimates to better reflect state-specific characteristics, we ran a logit model on the entire nonelderly female ACS PUMS sample (as opposed to only those in Massachusetts) using the same variables as in the Massachusetts-only model, but also adding dummy variables for each state and the District of Columbia, except Massachusetts. The coefficients for each state dummy variable capture the difference in likelihood of being uninsured due to residing in a given state relative to Massachusetts, after all individual-level characteristics specified in the regression are controlled. These state “fixed effects” thus measure variation in state-related insurance outcomes due to omitted variables, such as state health insurance market differences or other sociodemographic factors.

Next, we calibrated our model to correspond with other national estimates of the impact of the ACA. Based on estimates of the Congressional Budget Office (CBO)(6) and the Urban Institute (7), we inferred an estimate in the range of 9.6% to 10.0% uninsured for nonelderly adults (both men and women) of all incomes at the national level after reform. We calibrated our estimates to correspond to this target range using a partial adjustment of the results from the state fixed-effects estimates. Because they are derived using pre-health reform data, to use the entire fixed effect coefficient in estimating an individual’s likelihood of being uninsured would produce estimates that return the state’s 2009 level of uninsurance. Our model assumes that there is a partial adjustment from the state’s current level of insurance coverage toward the Massachusetts-based estimates. This assumption is based on the facts that health reform increases insurance coverage by creating minimum national standards but still offers states flexibility in policy design and implementation. We estimated the partial adjustment using a fraction (one-sixth) of the fixed effects coefficient, with the level corresponding to our calibration target.

We updated and aged our estimates from 2009 to 2014, using the Census Bureau’s national level projections of the age and size of the population (8). We adjusted the ACS-PUMS weights by factors associated with the expected change in the population by year of age.

After deriving a predicted probability of being uninsured for each individual in the data set, we computed mean predicted probabilities of being uninsured for each state using individual-level weights adjusted to reflect population growth between 2009 and 2014.

Finally, we computed state-specific estimates of the number of low-income women aged 18 to 64 where “low income” is defined using each state’s income criteria for the NBCCEDP program. We also produced estimates of the number of low-income women aged 40 to 64. The following income criteria applied: 185% FPL or below (Oklahoma), 200% FPL or below (Alabama, Arkansas, California, Connecticut, Florida, Georgia, Idaho, Indiana, Missouri, Montana, North Dakota, Ohio, South Carolina, South Dakota, Texas, Virginia, West Virginia), 226% FPL or below (Kansas, Nebraska), 250% FPL or below (all other states).

After deriving the number of people in each income group for each state, we used the predicted probabilities of being uninsured derived above to estimate state-by-state uninsured counts and rates for low income women. These counts of uninsured correspond to expected eligibility for the NBCCEDP eligibility groups: cervical cancer screening (women ages 18–64) and breast cancer screening (women ages 40–64). (Note: CDC is considering increasing the target age for cervical cancer screening to 21–64, based on recent changes in the US Preventive Services Task Force recommended age range.)

To derive estimates of the characteristics of the uninsured in 2014, we used the demographic data in the ACS, our 2014 expected population weights, and our predicted probabilities of being uninsured. First, we estimated the expected size of different population subgroups in 2014 using the ACS data and our 2014 weights (eg, Hispanic women, women without a high school degree or GED). We next used the predicted probability of being uninsured (assigned to all people in the ACS sample) to estimate uninsured rates among population subgroups, and subsequently, the number of uninsured in each population subgroup. Using the numbers of uninsured women in population subgroups, we estimated the percent of all uninsured women who were Hispanic, black, white, without a high school degree or GED, etc.

**Limitations of This Approach**

Our estimation approach differs significantly from those of the CBO (9) and the Urban Institute (10). Those models were designed as overall health policy simulation tools to estimate national-level effects under a wide range of assumptions for alternative versions of national health reform policies. They are complex simulations based on an amalgam of data sources, particularly the ASEC-CPS and the Medical Expenditure Panel Survey, and entail a large number of models and assumptions about behavioral responses to different policies (11). While these models are extraordinarily useful in estimating budget and other impacts of different policy choices, it has long been recognized that different models, and very slight differences in assumptions made, can have profound differences in the results, so that different models can yield very divergent results (12).

Our model used a much stronger sampling base and a simple, transparent set of assumptions. We used the ACS, which is a stronger base for state-level estimates because of its larger sample frame and more straightforward questions about health insurance status. And we assumed that the Massachusetts model can serve as a template for national estimates, with adjustments as we describe. To try to fit the national estimates, we — like the Urban Institute — calibrated our results to correspond approximately with the CBOs, so that everyone is working with similar national assumptions.

Like any forecasting model, our model is subject to flaws because we assumed that future behaviors and policies can be predicted based on behaviors and policies of the past. As noted earlier, our estimates correspond only to the non-institutionalized population in the 50 states and the District of Columbia. Although we made adjustments for national population growth and aging, we assumed that state patterns are similar to those projected for the nation. Our models also assumed that other characteristics in 2014, such as employment, income, and education levels, are similar to those of 2009. In 2009, the country was in a recession and unemployment levels were relatively high. If the economy strengthens by 2014, as we hope it will, uninsurance rates might be lower than estimated. On the other hand, if health care costs rise faster than anticipated (resulting a decreased propensity of individuals and firms to obtain health insurance), then uninsurance rates might be higher than estimated. Finally, if the ACA is not implemented in a fashion consistent with the expectations of the CBO, then the number of uninsured people may vary. While these limitations introduce significant uncertainty into our estimates, the sheer magnitude of the changes reported in our analyses imply that major changes are in store for the number and distribution of uninsured women in the United States after the implementation of health reform.

**References**

1. US Census Bureau. About health insurance. Washington (DC). <http://www.census.gov/hhes/www/hlthins/about/index.html>. Accessed April 26, 2011.

2. Turner J, Boudreaux M, Lynch V. A preliminary evaluation of health insurance coverage in the 2009 American Community Survey. Washington (DC): Health Policy Center, Urban Institute; 2009.

3. Fronstin P. Sources of health insurance and characteristics of the uninsured: analysis of the March 2010 Current Population Survey. **EBRI Issue Brief No. 347. Washington (DC): Employee Benefit Research Institute; 2010.**

**4. Ku L, Matani S. Left out: immigrants’ access to health care and insurance. Health Aff (Millwood) 2001;20(1):247–56.**

**5.** Table: medical assistance programs for immigrants in various states. <http://www.nilc.org/pubs/guideupdates/med-services-for-imms-in-states-2010-07-28.pdf>. Accessed May 23, 2011.

6. Congressional Budget Office. Final cost estimate, March 20, 2010. In: Selected CBO publications related to health care legislation, 2009–2010. Washington (DC): Congressional Budget Office; 2010. p. 3.

7. Buettgens M, Holahan J, Carrol C. Health reform across the states: increased insurance coverage and federal spending on the exchanges and Medicaid. Washington (DC): Urban Institute; 2011.

8. U.S. Census Bureau. US population projections. <http://www.census.gov/population/www/projections/summarytables.html>. Accessed April 26, 2011.

9. Congressional Budget Office. CBO’s health simulation model: a technical description. Washington (DC): Congressional Budget Office; 2007.

10. The Urban Institute’s health microsimulation capabilities. Washington (DC): Urban Institute; 2010. <http://www.urban.org/uploadedpdf/412154-Health-Microsimulation-Capabilities.pdf>. Accessed August 29, 2012.

11. Bilheimer L, Reischauer R. Confessions of the estimators: numbers and health reform. Health Aff (Millwood) 1995;14(1):37–55.

12. Nichols L. Perspectives: numerical estimates and the policy debate. Health Aff (Millwood) 1995;14(1):56–9.

**Table A1. Logit Results for Determinants of Female Uninsured Status in Massachusetts, Ages 18 to 64, 2009 American Community Survey^a^**

| **Variable** | **Odds Ratio (95% Confidence Interval)** |
| --- | --- |
| Age | 1.02 (0.97–1.08) |
| Age squared | 1.00 (1.00–1.00) |
| Hispanic | 1.02 (0.70–1.47) |
| Non-Hispanic African American | 0.44 (0.20–0.98) |
| Other race | 1.21 (0.87–1.68) |
| Noncitizen | 2.31 (1.77–3.01) |
| Income as % of poverty | 0.67 (0.61–0.73) |
| Income below poverty | 0.52 (0.37–0.71) |
| Works full-time full-year | 0.51 (0.38–0.70) |
| Works but not full-time full-year | 0.79 (0.60–1.04) |
| Self employed | 0.79 (0.51–1.23) |
| Not in the labor force | 0.89 (0.64–1.26) |
| Married | 0.59 (0.46–0.76) |
| Has child under age 7 y at home | 0.48 (0.35–0.66) |
| Has child aged 7 y to17 y at home | 0.56 (0.43–0.72) |
| Disabled | 0.65 (0.44–0.96) |
| Receives public income | 0.20 (0.12–0.35) |
| Graduated from college | 0.75 (0.58–0.97) |
| High school dropout | 1.18 (0.89–1.58) |
| Current student | 0.48 (0.34–0.68) |
| Works in high-insurance industry | 0.63 (0.42–0.93) |
| Works in low-insurance industry | 1.53 (1.20–1.95) |
| Over age 39/Hispanic interaction | 1.73 (1.05–2.88) |
| Black/income interaction | 1.35 (1.06–1.73) |
| Black/federal poverty level interaction | 1.94 (0.71–5.30) |

^a^ N = 20,548; model χ^2^_25_ = 625.8

**Table A2. Comparison of Actual Uninsured Rates to Predicted Uninsured Rates for Massachusetts Female Subgroups, 2009**

| **Population** | **Uninsured Actual Rate** | **Predicted Uninsured Rate** |
| --- | --- | --- |
| Women aged 18–64 | 0.0431 | 0.0431 |
| Hispanics aged 18–64 | 0.0931 | 0.0931 |
| Blacks aged 18-64 | 0.0624 | 0.0624 |
| Over age 39 | 0.0317 | 0.0324 |
| Hispanic over age39 | 0.0962 | 0.0962 |
| Black over age 39 | 0.0507 | 0.0514 |
| Income <201% poverty, aged18-64 | 0.0877 | 0.0936 |
| Hispanic, income <201% poverty, 18–64 | 0.1187 | 0.1139 |
| Black, income <201% poverty, aged 18–64 | 0.0703 | 0.0805 |
| Income <201% poverty, over age 39 | 0.0780 | 0.0782 |
| Hispanic, income <201% poverty, over age 39 | 0.1211 | 0.1187 |
| Black, income <201% poverty, over age 39 | 0.0693 | 0.0727 |
